# Fine mapping of *Ascaris lumbricoides, Trichuris trichiura* and hookworm infections in sub-districts of Makenene in Centre Region of Cameroun

**DOI:** 10.1038/s41598-022-18285-7

**Published:** 2022-08-17

**Authors:** Cyrille Nguemnang Kamdem, Auvaker Arnol Zebaze Tiofack, Estelle Mezajou Mewamba, Esthelline Yangea Tchounkeu, Joël Rostand Atiokeng Tatang, Edmond Loic Tekeu Mengoue, Carole Mureille Tchami Mbagnia, Pythagore Soubgwi Fogue, Hilaire Macaire Womeni, Gustave Simo

**Affiliations:** 1 Molecular Parasitology and Entomology Unit, Department of Biochemistry, Faculty of Science, University of Dschang, PO Box 67, Dschang, Cameroon; 2 Research Unit of Biology and Applied Ecology, Department of Animal Biology, Faculty of Science, University of Dschang, Dschang, Cameroon; 3 Parasitology and Ecology Laboratory, Department of Animal Biology and Physiology, Faculty of Science, University of Yaoundé I, Yaoundé, Cameroon; 4 “Unité de Recherche de Biochimie, des plantes Médicinales, des Sciences Alimentaires et Nutrition”, University of Dschang, Dschang, Cameroon

**Keywords:** Epidemiology, Translational research

## Abstract

Preventive chemotherapy (PC) that remains the main control strategy recommended by the World Health Organization to achieve the elimination of soil-transmitted helminth (STH) infections as a public health problem must be strengthened by identifying the remaining transmission hot-spots for the deployment of appropriate control measures. This study was designed to assess the prevalence and infections intensities of soil-transmitted helminths and perform micro scale mapping in order to identify transmission hot-spots for targeted control operations. Stool samples were collected from 1775 children in ten primary schools of eight sub-districts of Makenene in Cameroon. Kato Katz technique was used to process and examine stool samples to detect the eggs of soil-transmitted nematodes. The prevalence of soil-transmitted helminth species as well as the infection intensities was compared. Data visualizations in forms of maps were made using Quantum geographic information system (QGIS) software. The overall prevalence of soil-transmitted helminth infections was 4.8% with a 95% confidence interval (CI) of 3.8–5.9%: 3.0% (95% CI 2.2–3.9) for A*scaris lumbricoides*, 1.4% (95% CI 0.9–2.0) for *Trichuris trichiura* and 0.8% (95% CI 0.5–1.4) for hookworms. The prevalence of soil-transmitted helminth species differ significantly between schools and sub-districts. The intensity of infections was light (2.4%, 1.1% and 0.8%), moderate (0.4%, 0.1% and 0.1%) and heavy (0.2%, 0.2% and 0%) for A. *lumbricoides*, *T. trichiura* and hookworm respectively. The mean intensity of infections was 7255 EPG for *A. lumbricoides*, 2900 EPG for *T. trichiura* and 298 EPG for hookworm. Between schools, significant difference was recorded in the means of infection intensities of *T. Trichiura* and hookworms but not for *A. lumbricoides*. This difference was also significant for *T. Trichiura* when comparison were between sex. No significant difference were recorded when the comparison were between age*.* Fine mapping revealed that children harbouring heavy infections were clustered in the same sub-districts; highlighting the presence of high endemicity sub-districts and hot-spots for the transmission of different soil-transmitted helminth species. This study showed a diversity in the prevalence and transmission of different soil-transmitted helminth species. It also hightlighted the need for micro scale mapping to enable the localisation of high endemicity sub-districts and transmission hot-spot sites where targeted control operations must be deployed to achieve STH elimination.

## Introduction

Soil-transmitted helminth (STH) infections refers to a group of parasitic diseases caused by nematode worms that are transmitted to humans by the soil containing contaminated faeces. They are among the most widely distributed parasitic infections in tropical and subtropical countries^1^. Belonging to the 20 neglected tropical diseases listed by the World Health Organization (WHO), these parasitic diseases affect more than 1.5 billion people worldwide with the highest prevalence in Asia and Africa^2^. Over 267 million preschool-aged children (pre-SAC) and 568 million school-aged children (SAC) living in the least developed settings are primarily affected by these parasites^2^. *Ascaris lumbricoides* (roundworm), *Trichuris trichiura* (whipworm) and hookworm *(Necator americanus* and *Ancylostoma duodenale)* are the most predominant species among the group of soil-transmitted helminths. *Ascaris lumbricoides* infects about 820 million people while more than 450 million active cases of *T. trichiura* are estimated worldwide^2^. Globally, an estimated 450 million people have chronic hookworm infections^3^. The global burden of these parasitic diseases has been estimated at 1.97 million disability-adjusted life years (DALYs)^4^. In sub-Saharan Africa, it has been estimated that more than 30.7 million SAC were infected with *A. lumbricoides*, 36.5 million with *T. trichiura* and 50 million with hookworms^5^.

To overcome the morbidities related to STH, the WHO recommends preventive chemotherapy (PC) as a public health intervention to reduce the worm burden especially in pre-SAC, SAC, adolescent girls, women of the reproductive age group and adults in certain high-risk occupations^2^. Although PC and other supplementary interventions such as the improvement of water, sanitation and hygiene, change of behaviours through the dissemination of information and health education have reduced the prevalence and intensity of soil-transmitted helminth infections, the gain was spatiotemporally heterogeneous^6–11^. This suggests stringent monitoring and evaluation of the deworming process in different contexts^8,10^.

The morbidity elimination and transmission breaking targets of soil-transmitted helminth infections need consistent monitoring and evaluation of control programs to improve epidemiological knowledge and focus interventions. As most endemic areas under PC are moving from control towards the elimination target, fine mapping of soil-transmitted helminths and their infection intensities could help to understand the spatio-epidemiological differences for the improvement of control strategies. Up till now, STH transmission hot-spots have not been exhaustively identified^8,10^. In addition, monitoring the impact of existing interventions on the distribution of STH has been poorly addressed in areas presenting bio-ecological differences^12–16^. It is obvious that reaching WHO goal of eliminating STH requires to take into consideration the transmission hot-spots. In such context, there is therefore the need to update and fine map data on the prevalence and infection intensities of soil-transmitted helminth species in different epidemiological settings. This fine-mapping could provide significant contributions to understand the micro scale transmission of soil-transmitted helminths^17–19^.

This study was designed to assess the prevalence and infection intensities of soil-transmitted helminth species and perform fine scale mapping in order to identify hot-spot transmission sites and sub-districts of high endemicity for the deployment of targeted control operations.

## Materials and methods

### Ethical considerations

The protocol of this study was approved by the “National Ethical Committee for Research for Human Health” of the Ministry of Public health of Cameroon with the reference number N° 2018/05/1004/CE/CNERSH/SP that was renewed with the reference number N° 2019/07/1172/CE/CNERSH/SP. It was also approved by the review board of the Molecular Parasitology and Entomology Subunit of the Department of Biochemistry of the Faculty of Science of the University of Dschang. Before field surveys, approval was also obtained from administrative and traditional authorities, school inspectors, directors and teachers. Parents or guardians of participating children approved their participation by signing the informed consent form on their behalf. Children between ages 10 and 14 signed an assent form in addition to the signed informed consent form from their parent or guardians. In younger ones, only the signed consent form from their parents or guardians was considered. After detailed explanation of the objectives, the procedures and the potential risks and benefits, each child was free to choose whether to participate in the study or not. Only children who gave their assent and whose parents or guardians approved their participation by signing the consent form were sampled. During analyses, data of each child were anonymised.

Results of the parasitological analyses were communicated to parents or guardians and all children found with at least one species of soil-transmitted helminths were treated with Albendazole (single 400 mg dose) following WHO recommendations^20^.

### Study site

**Figure 1:**
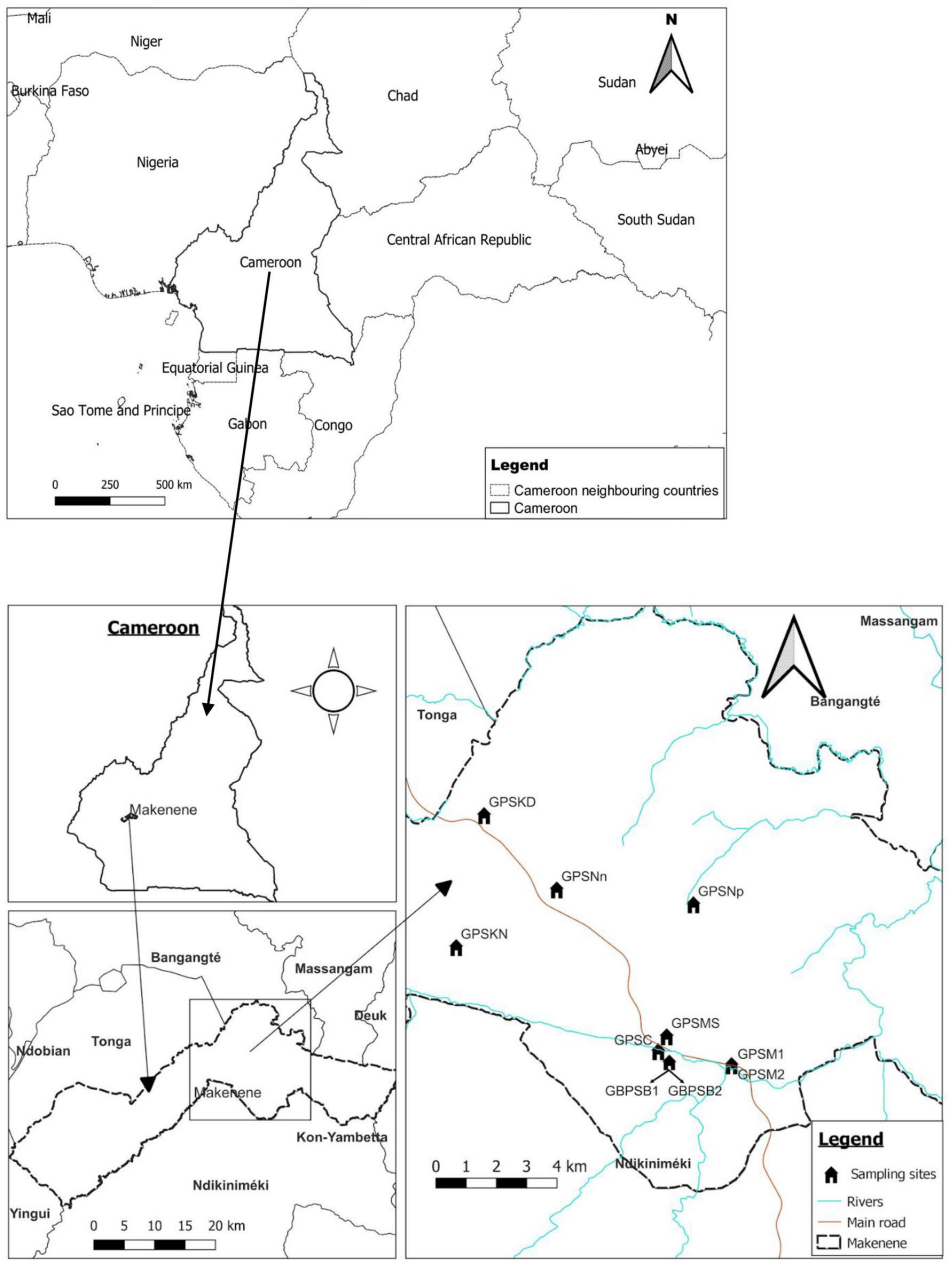
Map of Makenene showing schools where children were sampled (map obtained from: https://qgis.org/downloads/QGIS-OSGeo4W-3.16.10-1-Setup-x86_64.exe with layers downloaded using the link: https://www.diva-gis.org/gdata; consulted, 14/09/2021). GBPSB1: Government Bilingual Primary School of Baloua Group 1; GBPSB2: Government Bilingual Primary School of Baloua Group 2; GPSM1: Government Primary School of Makenene Group 1; GPSM2: Government Primary School of Makenene Group 2; GPSNn: Government Primary School of Nyokon; GPSKD: Government Primary School of Kinding Nde; GPSKN: Government Primary School of Kinding-Ndjabi; GPSMS: Government Primary School of Mock-Sud; GPSC: Government Primary School of Carrière; GPSNp: Government Primary School of Ngokop.

This study was conducted in December 2020 in 8 sub-districts of Makenene of “Mbam and Inoubou” Division of the Centre Region of Cameroon (Fig. 1). Makenene is located between latitude 4° 53′ 04″ north of the equator and longitude 10° 47′ 40″ East of the Greenwich Meridian, at an altitude of about 850 m above sea level. It covers about 885 km^2^ with an estimated population of about 16,000 inhabitants. The topography of the area is dominated by plains and valleys. The locality has two seasons: a short dry (November to March) and a long rainy season (March to November). It has a Guinean equatorial climate with an average rainfall of 1560 mm per year and an average temperature of 25 °C^21^. Its hydrographic network is made up of several rivers including Mock, Makombé, Makango, Managa, Mefom, Niep, Bakokeut, Kyakam, Mayi, Molo, Makam, Sinsan, Bambi, Djanka and Noum. Peasant agriculture is dominated by the culture of cassava, maize, groundnuts, yams, cocoa and palm nuts. Soil-transmitted helminth infections was reported in Makenene since more than three decades^12,22^. For more than ten years, this locality was under repeated and annual distribution of Mebendazole only to SAC. This distribution was performed by national control program of schistosomiasis and STH infections^14^. In this locality, neither Mebendazole nor Albendazole have been massively administered to inhabitants by another national control program against other infectious diseases.

#### Population and study design

Participants of this cross-sectional study were children attending ten primary schools of 8 sub-districts of Makenene (Fig. 1). Out of 14 schools of this locality, authorisations to conduct this study were obtained for 10 of them. These ten schools were located in 8 sub-districts of Makenene. In six sub-districts including Kinding Nde, Kinding Ndjabi, Nyokon, Carrière, Ngokop and Mock-sud, only one school was found and all the six were included in this study. However, in each sub-district of Makenene and Baloua, 4 primary schools were found due to the high population density compared to the other sub-districts. Authorisations to perform the study were obtained for the two public schools attended by the majority of SAC of these two sub-districts. For sampling, all children between 5 and 15 years attending the 10 schools were enrolled.

The sample size was made up of all SAC of 5–15 years attending the 10 primary schools of eight sub-districts and for whom a signed inform consent form was obtained from their parents or guardians.

### Collection of stool samples

Prior to stool collection, the administrative, traditional and religious authorities, school headmasters, teachers, parents and guardians of SAC were carefully informed. During the information process, the objective of the study was explained to directors and teachers of each school. The sampling day was communicated to children by their teachers. In the morning of each sampling day, orientations about the sampling process were given to directors and teachers before starting stool collection. Thereafter, each child who provided assent and had a signed inform consent from their parents or guardians was invited to provide stool sample in clean and well-labelled plastic containers that were given the day of sampling. Each plastic container was labelled on the field with the participant's unique identification number. Approximately 5–7 g of stool were collected from each child between 9:00 a.m. and 2:00 p.m. Stool samples were packed in a plastic bag and immediately transported (for less than 20 min) at ambient temperature (approximately 25 °C) to the nearest health Centre for parasitological analyses. Stool samples were kept at room temperature and their analysis were completely performed within 12 h following their collection.

### Laboratory processing and examination of stool samples

Each stool sample was processed to detect the eggs of soil-transmitted helminths using the Kato-katz (KK) technique as described by Katz et al*.*^23^. For each stool samples, one single KK thick smears slide was prepared by fixing 41.7 mg of stool on a punched template Sterlitech kit (Lot: XGACAI). Briefly, a small amount of stool sample was transferred onto a piece of scrap paper. Thereafter, a nylon mesh was pressed on the top of the faecal sample and a small plastic spatula was used to scrap the sieved material of the nylon screen. After placing the KK template on a clean microscopic slide, the well of the template was completely filled with sieved faecal material. The excess of sieved faecal material from the edge of the template hole was carefully removed. The template was then removed without disturbing the calibrated faecal material. Then, the slide was covered with a cellophane strip pre-soaked for 24 h in glycerol-malachite green solution. The stool was spread onto a thick smear by spreading the stool against the cellophane paper with an even-edged spatula. The slides were then observed within less than three hours under a light microscope at 10× magnification. When a slide was positive, the magnification was increased to 40× for better observation of the eggs. Eggs of each species of soil-transmitted helminths were morphologically identified by examining each microscope slide. For each slide that was positive for at least one soil-transmitted helminth species, the eggs of each species were counted and the intensity of infection estimated in eggs per gram (EPG) of stools as described by Montresor et al*.*^24^. After this estimation, the infection intensities were classified as light, moderate or heavy^24^. For *A. lumbricoides*, the values of infection intensities classified as light, moderate or heavy were respectively 1 EPG to 4999 EPG, 5000 EPG to 49,999 EPG and ≥ 50,000 EPG while for *T. trichiura,* these values were 1 EPG to 999 EPG for light infection intensity, 1000 EPG to 9999 EPG for moderate and ≥ 10,000 for EPG heavy. Regarding hookworm, the infection intensity of 1 to 1999 EPG was classified as light while those of 2000 EPG to 3999 EPG and ≥ 4000 EPG were respectively classified as moderate and heavy.

### Collection of geographical coordinates of children's residences carrying soil-transmitted helminths

After performing KK test on all stool samples, children harbouring soil-transmitted helminth infections were identified. Thereafter, each infected child returning home went with at least one member of the research team. Once reaching the home of infected child, the geographical coordinates of the residence of this child were recorded using a Global Position System device (eTrex, Garmin International, Olathe, KS, USA). Recording of residences’ coordinates was performed after detailed explanation of the purpose of the study and the approval of the head of the family. Other factors that could influence with STH transmission such as the presence of borehole water, wells and toilet (clean or with poor hygienic conditions) were recorded.

### Data analysis

Cameroon map containing administrative divisions was downloaded using the link https://www.diva-gis.org/gdata. Thereafter, the geographical information layers on the road and hydrographic networks were projected onto the map using the geographical information system software (QGIS v.3.16.10 'Hannover'; QGIS Development Team, https://qgis.org/downloads/QGIS-OSGeo4W-3.16.10-1-Setup-x86_64.exe). The geographical coordinates of schools, residences of infected children and some environmental factors (borehole water, wells and toilet) that could influence with STH transmission were inserted onto the map using the geographical information system software (QGIS v.3.16.10 'Hannover'; QGIS Development Team, https://qgis.org/downloads/QGIS-OSGeo4W-3.16.10-1-Setup-x86_64.exe). This software was then used to map the infection of each soil-transmitted helminth species as well as its infection intensities.

Chi-square test was used to compare the prevalence of soil-transmitted helminth species between sub-districts, schools, ages and sexes. The Kolmogorov–Smirnov test was used to check if the data of the infection intensities of STH were normally distributed. Following this test, the Mann Whitney U and Kruskal Wallis H tests were used to compare the geometric and arithmetic means of infection intensities between schools, ages and sexes. The test was considered significant when the p-value was below 0.05.

### Institutional Review Board Statement

The study was conducted according to the guidelines of the Declaration of Helsinki, and approved by the “National Ethical Committee for Research for Human Health” of the Ministry of Public health of Cameroon with the reference number N° 2018/05/1004/CE/CNERSH/SP delivered on 4 May 2018, that has been renewed with the reference number N° 2019/07/1172/CE/CNERSH/SP delivered on 12 July 2019. It was also approved by the review board of the Molecular Parasitology and Entomology Subunit of the Department of Biochemistry of the Faculty of Science of the University of Dschang.

### Informed consent statement

Informed consent was obtained from all subjects involved in the study.

## Results

### Characteristics of the study population

A total of 1775 stool samples were collected from 947 (53.4%) boys and 828 (46.6%) girls (Table 1). The mean age of children examined was 8.53 ± 2.21 (95% CI 8.40–8.67) years: 8.70 ± 2.34 (95% CI 8.51–8.89) for boys and 8.34 ± 2.04 (95% CI 8.14–8.56) for girls (Table 2). Boys were significantly older than girls (*t*_*(1773)*_ = *3.46, p* = *0.001*). These children attended ten primary schools located in eight sub-districts of the locality of Makenene (Table 3).

**Table 1:** Prevalence and infection intensities of soil-transmitted helminth species according to sex.

Sex	NSA	STHs combined		*A. lumbricoides*					*T. trichiura*					Hookworm				
		KK (P)	GM & AM^#^ in EPG (± SD)	LI	MI	HI	P	GM & AM^#^ in EPG (± SD)	LI	MI	HI	P	GM & AM^#^ in EPG (± SD)	LI	MI	HI	P	GM & AM^#^ in EPG (± SD)
Male	947	44 (4.6)	188 (± 26,491), 8422 (± 26,491)^#^	21	2	3	2.7	308 (± 28,509), 11,577 (± 28,509)^#^	10	2	3	1.6	461 (± 7343), 4589 (± 7343)^#^	8	0	0	0.8	48 (± 115), 90 (± 115)^#£^
Female	828	41 (5.0)	102 (± 5735), 2148 (± 5735)^#^	22	5	0	3.3	130 (± 6837), 3094 (± 6837)^#^	9	0	0	1.1	62 (± 82), 86 (± 82)^#^	6	1	0	0.8	73 (± 1172)*, 534 (± 1172)^#^
Total	1775	85 (4.8)	140 (± 19,618), 5395 (± 19,618)^#^	43	7	3	3.0	198 (± 20,795), 7255 (± 20,795)^#^	19	2	3	1.4	217 (± 6147), 2900 (± 6147)^#^	14	1	0	0.8	59 (± 805), 298 (± 805)^#^
^**.**^**p-value**		**0.76**^**a**^	**0.17**^**b**^				**0.53**^**a**^	**0.16**^**b**^				**0.37**^**a**^	**0.04**^**b**^				**1**^**a**^	**0.95**^**b**^
**(**χ^**2**^**)**		**0.09**					**0.41**					**0.82**					**0.000**	
**z**			**1.37**					**1.40**					**2.02**					**0.07**

*STHs* soil transmitted helminths; *A. lumbricoides*, *Ascaris lumbricoides*; *T. trichiura*, *Trichuris trichuria*; *NSA* number of stool samples analysed; *KK* overall number of children positive for the kato katz; *GM* geometric mean egg count; *AM*^*#*^ arithmetic mean egg count; *SD* standard deviation; *LI* number of children having light infection; *MI *number of children having moderate infection; *HI* number of children having heavy infection; *EPG* egg per gram of stool; *P* prevalence.

Significant values are in [bold].

*Two co-infections.

^£^Five co-infections.

^a^p-value for chi-square test.

^b^p-value for Mann Whitney U test.

**Table 2:** Prevalence and infection intensities of soil-transmitted helminth species according to age.

Age	NSA	STHs combined		*A. lumbricoides*					*T. trichiura*					Hookworm				
		KK (P)	GM & AM^#^ in EPG (± SD)	LI	MI	HI	P	GM & AM^#^ in EPG (± SD)	LI	MI	HI	P	GM & AM^#^ in EPG (± SD)	LI	MI	HI	P	GM & AM^#^ in EPG (± SD)
5	85	5(5.9)	114 (± 1393), 677 (± 1393)^#^	4	0	0	4.7	45 (± 20), 48 (± 20)^#^	0	0	0	0	0 (–), 0 (–)^#^	1	1	0	2.4	275(± 2223), 1596 (± 2223)^#^*
6	347	16(4.6)	71 (± 15,084), 3845 (± 15,084)^#^	10	0	1	3.2	92 (± 18,188), 5571 (± 18,188)^#^	3	0	0	0.9	55 (± 37), 64 (± 37)^#^	2	0	0	0.6	24 (± 0.00), 24 (± 0.00)^#^
7	225	8(3.6)	266 (± 8775), 4776 (± 8775)^#^	3	2	0	2.2	982 (± 10,380), 7623 (± 10,380)^#^	0	0	0	0	0 (–), 0 (–)^#^	3	0	0	1.3	30 (± 14), 32 (± 14)^#^
8	264	14(5.3)	166 (± 28,710), 10,608 (± 28,710)^#^	6	1	1	3.0	213 (± 29,711), 13,368 (± 29,711)^#^	3	1	2	2.3	938 (± 8693), 6920 (± 8693)^#^	2	0	0	0.8	24 (± 0.00), 24 (± 0.00)^#£^
9	233	10(4.3)	151 (± 9893), 4755 (± 9893)^#^	6	2	0	3.4	227 (± 9222), 4989 (± 9222)^#^	2	1	0	1.3	160 (± 4101), 2392 (± 4101)^#^	1	0	0	0.4	456 (–), 456 (–)^#£^
10	273	10(3.7)	178 (± 40,899), 13,107 (± 40,899)^#^	4	0	1	1.8	179 (± 49,480), 22,176 (± 49,480)^#^	5	0	1	2.2	374 (± 7575), 3364 (± 7575)^#^	0	0	0	0	0 (–), 0 (–)^#^*
11	162	12(7.4)	215 (± 4212), 2094 (± 4212)^#^	6	2	0	4.9	413 (± 4948), 3081 (± 4948)^#^	3	0	0	1.9	55 (± 37), 64 (± 37)^#^	2	0	0	1.2	79(± 170), 144 (± 170)^#^*
12	114	6(5.3)	58 (± 386), 200 (± 386)^#^	2	0	0	1.8	154 (± 679), 504 (± 679)^#^	2	0	0	1.8	54 (± 68), 72 (± 68)^#^	2	0	0	1.8	24 (± 0.00), 24 (± 0.00)^#^
13	46	3(6.5)	219 (± 794), 608 (± 794)^#^	2	0	0	4.3	190 (± 1052), 768 (± 1052)^#^	0	0	0	0	0 (–), 0 (–)^#^	1	0	0	2.2	288 (–), 288 (–)^#^
14	17	0(0)	0 (–), 0 (–)	0	0	0	0	0 (–), 0 (–)^#^	0	0	0	0	0 (–), 0 (–)^#^	0	0	0	0	0 (–), 0 (–)^#^
15	9	1(11.1)	192 (–), 192 (–)	0	0	0	0	0 (–), 0 (–)^#^	1	0	0	11.1	192 (–), 192 (–)^#^	0	0	0	0	0 (–), 0 (–)^#^
Total	1775	85 (4.8)	140 (± 19,618)	43	7	3	3.0	198 (± 20,795), 7255 (± 20,795)^#^	19	2	3	1.4	217 (± 6147)	14	1	0	0.8	59 (± 805), 298 (± 805)^#^
**df**		**10**	**9**				**10**	**8**				**10**	**6**				**10**	**7**
**p-value**		**0.77**^**a**^	**0.74**^**b**^				**0.76**^**a**^	**0.63**^**b**^				**0.11**^**a**^	**0.42**^**b**^				**0.56**^**a**^	**0.34**^**b**^
**(χ**^**2**^**)**		**6.48**	**5.96**				**6.60**	**6.17**				**15.75**	**5.99**				**8.69**	**7.87**

*STHs* soil transmitted helminths; *A. lumbricoides*, *Ascaris lumbricoides*; *T. trichiura*, *Trichuris trichuria*; *NSA* number of stool samples analysed; *KK* overall number of children positive for the kato katz; *GM* geometric mean egg count; *AM*^*#*^ arithmetic mean egg count; *SD* standard deviation; *LI* number of children having light infection; *MI *number of children having moderate infection; *HI* number of children having heavy infection; *EPG* egg per gram of stool, *P* prevalence; *df* degree of freedom.

Significant values are in [bold].

*One co-infection.

^£^Two co-infections.

^a^p-value for chi-square test.

^b^p-value for Kruskal Wallis H test.

**Table 3:** Prevalence and infection intensities of soil-transmitted helminth species according to schools.

School	NSA	STHs combined		*A. lumbricoides*					*T. trichiura*					Hookworm				
		KK (P)	GM & AM^#^ in EPG (± SD)	LI	MI	HI	P	GM & AM^#^ in EPG (± SD)	LI	MI	HI	P	GM & AM^#^ in EPG (± SD)	LI	MI	HI	P	GM & AM^#^ in EPG (± SD)
GBPSB1	193	0(0)	0 (–), 0 (–)^#^	0	0	0	0	0 (–), 0 (–)^#^	0	0	0	0	0 (–), 0 (–)^#^	0	0	0	0	0 (–), 0 (–)^#^
GBPSB2	227	8(3.5)	289 (± 7957), 3516 (± 7957) ^#^	6	1	0	3.1	205 (± 84), 3501 (± 8421) ^#^	0	0	0	0	0 (–), 0 (–)^#^	1	1	0	0.9	1202 (± 1918), 1812 (± 1918)^#^*
GPSM1	139	4(2.9)	111(± 5400), 2724 (± 5400) ^#^	1	1	0	1.4	510 (± 7637), 5424 (± 7637) ^#^	1	0	0	0.7	24 (–), 24 (–)^#^	1	0	0	0.7	24 (–), 24 (–)^#^
GPSM2	155	6(3.9)	174 (± 9762), 4100 (± 9762) ^#^	2	1	0	1.9	538 (± 9639), 5768 (± 9639) ^#^	3	1	0	2.6	172 (± 3536), 1824 (± 3536) ^#^	0	0	0	0	0 (–), 0 (–)^#^*
GPSNn	174	15(8.6)	154 (± 6632), 2709 (± 6632)^#^	3	2	0	2.9	828 (± 10,153), 7910 (± 10,153)^#^	8	0	0	4.6	101 (± 86), 126 (± 86)^#^	3	0	0	1.7	24 (0.00), 24 (0.00)^#^*
GPSKN	141	8(5.7)	30 (± 18), 33 (± 18)^#^	4	0	0	2.8	34 (± 14), 36 (± 14)^#^	1	0	0	0.7	24 (–), 24 (–)^#^	4	0	0	2.8	24 (0.00), 24 (0.00)^#^*
GPSKD	80	6(7.5)	79 (± 380), 212 (± 380)^#^	3	0	0	3.8	166 (± 527), 376 (± 527)^#^	3	0	0	3.8	38 (± 42), 48 (± 42)^#^	0	0	0	0	0 (–), 0 (–)^#^
GPSMS	77	4(5.2)	29 (± 12), 30 (± 12)^#^	2	0	0	2.6	34 (± 17), 36 (± 17)^#^	0	0	0	0	0 (–), 0 (–)^#^	2	0	0	2.6	24 (0.00), 24 (0.00)^#^
GPSC	274	26(9.5)	284 (± 33,611), 13,114 (± 33,611)^#^	15	1	3	6.9	264 (± 32,835), 14,697 (± 32,835)^#^	3	1	3	2.6	2343 (± 9004), 8730 (± 9004)^#^	3	0	0	1.1	154 (± 132), 200 (± 132)^#£^
GPSNp	315	8(2.5)	87 (± 3949), 1464 (± 3949)^#^	7	1	0	2.5	87 (± 3948), 1464 (± 3948)^#^	0	0	0	0	0 (–), 0 (–)^#^	0	0	0	0	0 (–), 0 (–)^#^
Total	1775	85(4.8)	140 (± 19,618), 5395 (± 19,618)^#^	43	7	3	3.0	198 (± 20,795), 7255 (± 20,795)^#^	19	2	3	1.4	217 (± 6147), 2900 (± 6147)^#^	14	1	0	0.8	59 (± 805), 298 (± 805)^#^
**df**		**9**	**8**				**9**	**8**				**9**	**5**				**9**	**5**
**p-value**		**˂ 0.0001**^**a**^	**0.10**^**b**^				**0.006**^**a**^	**0.77**^**b**^				**˂ 0.0001**^**a**^	**0.04**^**b**^				**0.04**^**a**^	**0.01**^**b**^
**(χ**^**2**^**)**		**35.85**	**13.30**				**22.87**	**4.87**				**33.90**	**11.90**				**17.67**	**13.82**

*STHs* soil transmitted helminths; *A. lumbricoides*, *Ascaris lumbricoides*; *T. trichiura*, *Trichuris trichuria*; *GBPSB1* Government Bilingual Primary School of Baloua Group 1; *GBPSB2* Government Bilingual Primary School of Baloua Group 2; *GPSM1* Government Primary School of Makenene Group 1; *GPSM2* Government Primary School of Makenene Group 2; *GPSNn* Government Primary School of Nyokon; *GPSKD* Government Primary School of Kinding Nde; *GPSKN* Government Primary School of Kinding-Ndjiabi; *GPSMS* Government Primary School of Mock-Sud; *GPSC* Government Primary School of Carrière; *GPSNp* Government Primary School of Ngokop; *NSA* Number of stool samples analysed; *KK* Overall number of children positive for the kato katz, *GM* geometric mean egg count, *AM*^*#*^ arithmetic mean egg count, *SD* standard deviation, *LI* Number of children having light infection, *MI* Number of children having moderate infection, *HI* Number of children having heavy infection, *EPG* egg per gram of stool, *P* prevalence, *df* degree of freedom.

Significant values are in [bold].

^a^p-value for chi-square test.

^b^p-value for Kruskal Wallis H test.

*One co-infection.

^£^Three co-infections.

### Prevalence of soil-transmitted helminth species

Out of 1775 children from whom stool samples were collected and examined, 85 were infected by at least one soil-transmitted helminth species; giving an overall prevalence of 4.8% (95% CI 3.8–5.9) (Table 1). Eggs of three species of soil-transmitted helminths including *A. lumbricoides*, *T. trichiura* and hookworm were detected in stool samples. *A. lumbricoides* was the predominant species with the highest prevalence of 3.0% (95% CI 2.2–3.9), followed by *T. trichiura* and hookworm with respective prevalence of 1.4% (95% CI 0.9–2.0) and 0.8% (95% CI 0.5–1.4) (Tables 1, 2, and 3).

#### Prevalence of soil-transmitted helminth species according to sex and age

Comparing the overall prevalence of soil-transmitted helminths, no significant difference (*χ*^2^ = *0.09; df* = *1; p* = *0.76*) was observed between boys and girls. When this comparison was performed according to each soil-transmitted helminth species, no significant difference was also observed between boys and girls for *A. lumbricoides *(*χ*^2^ = *0.41; df* = *1; p* = *0.53*),* T. trichiura*: (*χ*^2^ = *0.82; df* = *1; p* = *0.37*) and hookworm (*χ*^2^ = *0.00; df* = *1; p* = *1*) (Table 1).

The overall prevalence of soil-transmitted helminths did not also differ (*χ*^2^ = *6.48; df* = *10; p* = *0.77*) according to ages. Regarding each soil-transmitted helminth species, no significant difference was recorded for *A. lumbricoides *(*χ*^2^ = *6.60; df* = *10; p* = *0.76*) *T. trichiura *(*χ*^2^ = *15.75; df* = *10; p* = *0.11*) and hookworm (*χ*^2^ = *8.69; df* = *10; p* = *0.56*) (Table 2).

#### Prevalence of soil-transmitted helminth species according to schools

The highest overall soil-transmitted helminths prevalence of 9.5% was recorded in SAC from the Public School of Carriere while no egg (no soil-transmitted helminth infection) was found in those from Government Bilingual Primary School of Baloua Group 1 (Table 3). The overall prevalence of soil-transmitted helminths differs significantly (*χ*^2^ = *35.85; df* = *9; p ˂0.0001*) between schools. Significant differences were recorded between schools for the prevalence of *A. lumbricoides (χ*^2^ = *22.87; df* = *9; P* = *0.006*),* T. trichiura (χ*^2^ = *33.90; df* = *9; p ˂ 0.0001*) and hookworm *(χ*^2^ = *17.67; df* = *9; p* = *0.04*) (Table 3).

#### Prevalence of soil-transmitted helminth species according to sub-districts

The highest overall soil-transmitted helminths prevalence of 9.5% was recorded at Carrière and the lowest value of 1.9% at Baloua (Table 4). Comparing the overall prevalence of soil-transmitted helminths, significance difference *(χ*^2^ = *41.80; df* = *7; p ˂ 0.0001)* was recorded between sub-districts (Table 4). When these comparisons were performed according to soil-transmitted helmimths species, significant differences were also recorded for *A. lumbricoides (χ*^2^ = *20.69; df* = *7; p* = *0.004)*, *T. trichiura (χ*^2^ = *28.75; df* = *7; p* = *0.0002)* and hookworm *(χ*^2^ = *26.56; df* = *10; p* = *0.0004)* (Table 4).

**Table 4:** Prevalence and infection intensities of soil-transmitted helminth species according to sub-districts.

Sub-district	NSA	STHs combined		*A. lumbricoides*					*T. trichiura*					Hookworm				
		KK (P)	GM & AM^#^ in EPG (± SD)	LI	MI	HI	P	GM & AM^#^ in EPG (± SD)	LI	MI	HI	P	GM & AM^#^ in EPG (± SD)	LI	MI	HI	P	GM & AM^#^ in EPG (± SD)
Baloua	420	8 (1.9)	289 (± 7957), 3516 (± 7957)^#^	6	1	0	1.7	205 (± 8421), 3501 (± 8421)^#^	0	0	0	0	0 (-), 0 (-)^#^	1	1	0	0.5	1202 (± 1918), 1812 (± 1918)^#^*
Makenene	294	10(3.4)	145 (± 7948), 3550 (± 7948)^#^	3	2	0	1.7	526 (± 7815), 5631 (± 7815)^#^	4	1	0	1.7	116 (± 3166), 1464 (± 3166)^#^	1	0	0	0.3	24 (-), 24 (-)^#^*
Nyokon	174	15(8.6)	154 (± 6632), 2709 (± 6632)^#^	3	2	0	2.9	828 (± 10,153), 7910 (± 10,153)^#^	8	0	0	4.6	101 (± 86), 126 (± 86)^#^	3	0	0	1.7	24 (± 0.00), 24 (± 0.00)^#^*
K. Ndjiabi	141	8(5.7)	30 (± 18), 33 (± 18)^#^	4	0	0	2.8	34 (± 14), 36 (± 14)^#^	1	0	0	0.7	24 (-), 24 (-)^#^	4	0	0	2.8	24 (± 0.00), 24 (± 0.00)^#^*
K. Nde	80	6(7.5)	79 (± 380), 212 (± 380)^#^	3	0	0	3.8	166 (± 527), 376 (± 527)^#^	3	0	0	3.8	48 (± 42), 48 (± 42)^#^	0	0	0	0	0 (-), 0 (-)^#^
Mock-Sud	77	4(5.2)	29 (± 12), 30 (± 12)^#^	2	0	0	2.6	34 (± 17), 36 (± 17)^#^	0	0	0	0	0 (-), 0 (-)^#^	2	0	0	2.6	24 (± 0.00), 24 (± 0.00)^#^
Carrière	274	26(9.5)	284 (± 33,611), 13,114 (± 33,611)^#^	15	1	3	6.9	264 (± 32,835), 14,697 (± 32,835)^#^	3	1	3	2.6	2343 (± 9004), 8730 (± 9004)^#^	3	0	0	1.1	154 (± 132), 200 (± 132)^#£^
Ngokop	315	8(2.5)	87 (± 3948), 1464 (± 3948)^#^	7	1	0	2.5	87 (± 3948), 1464 (± 3948)^#^	0	0	0	0	0 (-), 0 (-)^#^	0	0	0	0	0 (-), 0 (-)^#^
Total	1775	85(4.8)	140 (± 19,618), 5395 (± 19,618)^#^	43	7	3	3.0	198 (± 20,795), 7255 (± 10,153)^#^	19	2	3	1.4	217 (± 6147), 2900 (± 6147)^#^	14	1	0	0.8	59 (± 805), 298 (± 805)^#^
**df**		**7**	**84**				**7**	**7**				**7**	**4**				**7**	**5**
**p-value**		**˂ 0.0001**^**a**^	**0.08**^**b**^				**0.004**^**a**^	**0.69**^**b**^				**0.0002**^**a**^	**0.03**^**b**^				**0.0004**^**a**^	**0.01**^**b**^
**(χ**^***2***^**)**		**41.82**	**12.48**				**20.69**	**4.73**				**28.75**	**11.07**				**26.56**	**13.82**

*STHs* soil transmitted helminths; *A. lumbricoides*, *Ascaris lumbricoides*; *T. trichiura*, *Trichuris trichuria*; *K. Nde* Kinding Nde; *K. Ndjiabi* Kinding-Ndjiabi; *NSA* number of stool samples analysed; *KK* overall number of children positive for the kato katz; *GM* geometric mean egg count; *AM*^*#*^ arithmetic mean egg count; *SD* standard deviation; *LI* number of children having light infection; *MI* number of children having moderate infection; *HI* number of children having heavy infection; *EPG* egg per gram of stool; *P* prevalence; *df* degree of freedom.

Significant values are in [bold].

*One co-infection.

^£^Three co-infections.

^a^p-value for chi-square test.

^b^p-value for Kruskal Wallis H test.

### Infection intensity of soil-transmitted helminth species

*Ascaris lumbricoides* had the highest mean number of eggs per gram of stool (198 ± 20,795 and 7255 ± 20,795 EPG for geometric and arithmetic, respectively), followed by *T. trichiura* (217 ± 6147 and 2900 ± 6147 EPG for geometric and arithmetic, respectively) and then hookworm (59 ± 805 and 298 ± 805 for geometric and arithmetic, respectively) EPG. Comparing the means (geometric and arithmetic) of infection intensities, no significant difference was observed between boys and girls for *A. lumbricoides (z* = *1.40; p* = *0.16)* and hookworm *(z* = *0.07; p* = *0.95).* However, significant difference *(z* = *2.02; p* = *0.04)* was recorded *for T. trichiura* (Table 1)*.* When these comparisons were performed according to ages, no significant difference was recorded for *A. lumbricoides (χ*^2^ = *6.17; df* = *8; p* = *0.63), T. Trichiura (χ*^2^ = *5.99; df* = *6; p* = *0.42)* and hookworm *(χ*^2^ = *7.87; df* = *7; p* = *0.34)* (Table 2).

Between schools, significant difference was recorded for the means of infection intensities for *T. Trichiura (χ*^2^ = *11.90; df* = *5; p* = *0.04)* and hookworm *(χ*^2^ = *13.82; df* = *5; p* = *0.01)*. However, no significant difference was recorded for *A. lumbricoides (χ*^2^ = *4.87; df* = *8; p* = *0.77)* (Table 3).

From 53 children carrying *A. lumbricoides* eggs, 43 (2.4%: 53/1775) had light infection intensity while 7 (0.4%: 7/1775) and 3 (0.2%: 3/1775) had respectively moderate and heavy infection intensities. Out of 24 children carrying *T. Trichiura* eggs*,* 19 (1.1%: 19/1775) had light infection intensities whereas 2 (0.1%: 2/1775) and 3 (0.2%: 3/1775) had respectively moderate and heavy infections. For the 15 children carrying hookworm eggs, 14 (0.8%: 14/1775) and one (0.1%: 1/1775) had respectively light and moderate infection intensities.

Heavy infection intensities of *A. lumbricoides* and *T. trichiura* were found in boys from Public school of Carrière (Table 1). From 9 children carrying moderate infection intensities, five girls and 2 boys harboured *A. lumbricoides* while two (0.1%) other boys had *T. trichiura.* One girl with moderate infection intensities of *A. lumbricoides* harboured also moderate infection of hookworm (Table 1). All children harbouring moderate infection intensities were from six schools (GPSNn, GPSM 1, GBPSB 2, GPSC, GPSM2 and GPSNp) and from five sub-districts notably Ngokop, Carriere, Nyokon, Baloua and Makenene (Tables 3 and 4). They were 5 and 11 years old (Table 2).

Among the 43 children found with light infection intensities for *A. lumbricoides*, 21 (1.2%) were boys and 22 (1.2%) girls. From the 19 children harbouring light infection intensities for *T. trichiura,* 10 (0.6%) were boys and 9 (0.5%) were girls. Eight (0.5%) boys and 6 (0.3%) girls harboured light infection intensities for hookworm (Table 1). Children harbouring light infection intensities of different species of soil-transmitted helminths were between 5 and 15 years and were from all sub-districts (Table 2).

### Co-infections of different species of soil-transmitted helminths

From the 85 children carrying soil-transmitted helminths’ eggs, 78 were infected by one soil-transmitted helminth species while 7 had co-infections of different soil-transmitted helminth species. These 7 co-infections included 5 with *A. lumbricoides* and *T. trichiura* and the two with *A. lumbricoides* and hookworm. Three co-infections involving *A. lumbricoides* and *T. trichiura* were found in children of the Government Primary School of Carriere while the two other were recorded in children of Government Primary School of Makenene Group 2 and the Government Primary School of Nyokon. One co-infection involving *A. lumbricoides* and hookworm was recorded in a child of the Government Primary School Group 2 of Baloua while the other was in a child of Government Primary School of Kinding-Ndjabi (Table 3).

From the seven coinfected children, two harboured heavy infections for both *A. lumbricoides* and *T. trichiura* while two other had light infections: one involving *A. lumbricoides* and *T. Trichiura,* and the other involving *A. lumbricoides* and hookworm. One child had moderate infection intensities for both *A. lumbricoides* and *T. Trichiura*. Another had heavy and moderate infection intensities for *A. lumbricoides* and *T. trichiura* respectively*.* The last child had moderate infection intensity for *A. lumbricoides* and light intensity of infection for hookworm.

### Spatial distribution of different species of soil-transmitted helminths

**Figure 2:**
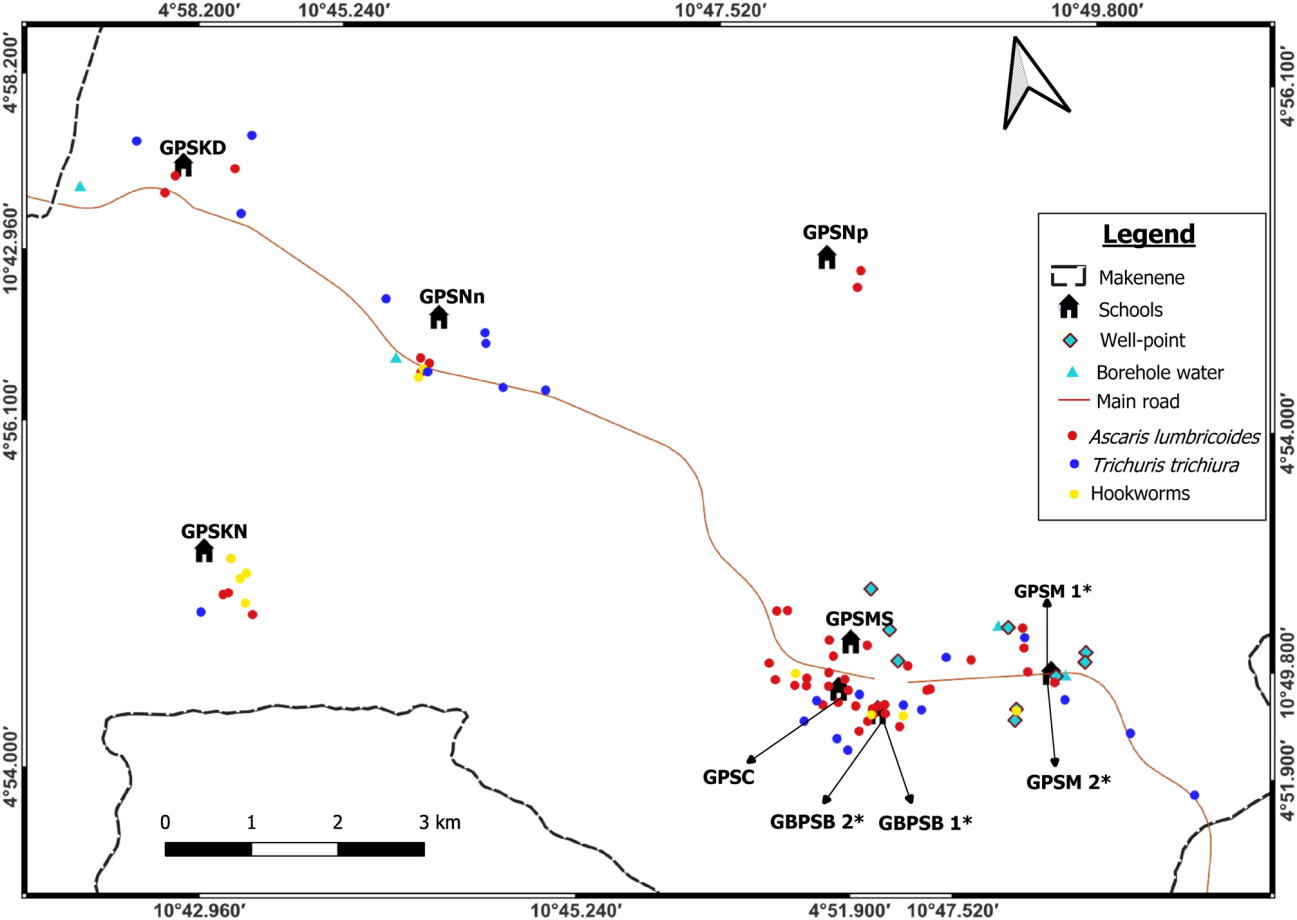
Map showing spatial distribution of different species of soil-transmitted helminths (*both schools are hosted on the same building). GBPSB1: Government Bilingual Primary School of Baloua Group 1; GBPSB2: Government Bilingual Primary School of Baloua Group 2; GPSM1: Government Primary School of Makenene Group 1; GPSM2: Government Primary School of Makenene Group 2; GPSNn: Government Primary School of Nyokon; GPSKD: Government Primary School of Kinding Nde; GPSKN: Government Primary School of Kinding-Ndjabi; GPSMS: Government Primary School of Mock-Sud; GPSC: Government Primary School of Carrière; GPSNp: Government Primary School of Ngokop.

Spatial distribution of soil-transmitted helminth infections shows that residences of infected children seem to cluster. Children harbouring soil-transmitted helminth infections were concentrated around the Government Primary School of Carriere, Government Primary School of Mock Sud and Government Bilingual Primary School of Baloua” Groups 1 and 2 (Fig. 2). Endemic sub-districts can be grouped into low, moderate and high endemicity. The highest concentration of children carrying soil-transmitted helminth infections was recorded in sub-districts Carriere, Mock-Sud and Baloua. These sub-districts can be considered of high endemicity for STH (Fig. 2). Sub-districts like Ngokop and Kinding-Nde are of low endemicity while Kinding-Ndjabi, Nyokon and Makenene are of moderate endemicity for soil-transmitted helminthiasis (Fig. 2).

**Figure 3:**
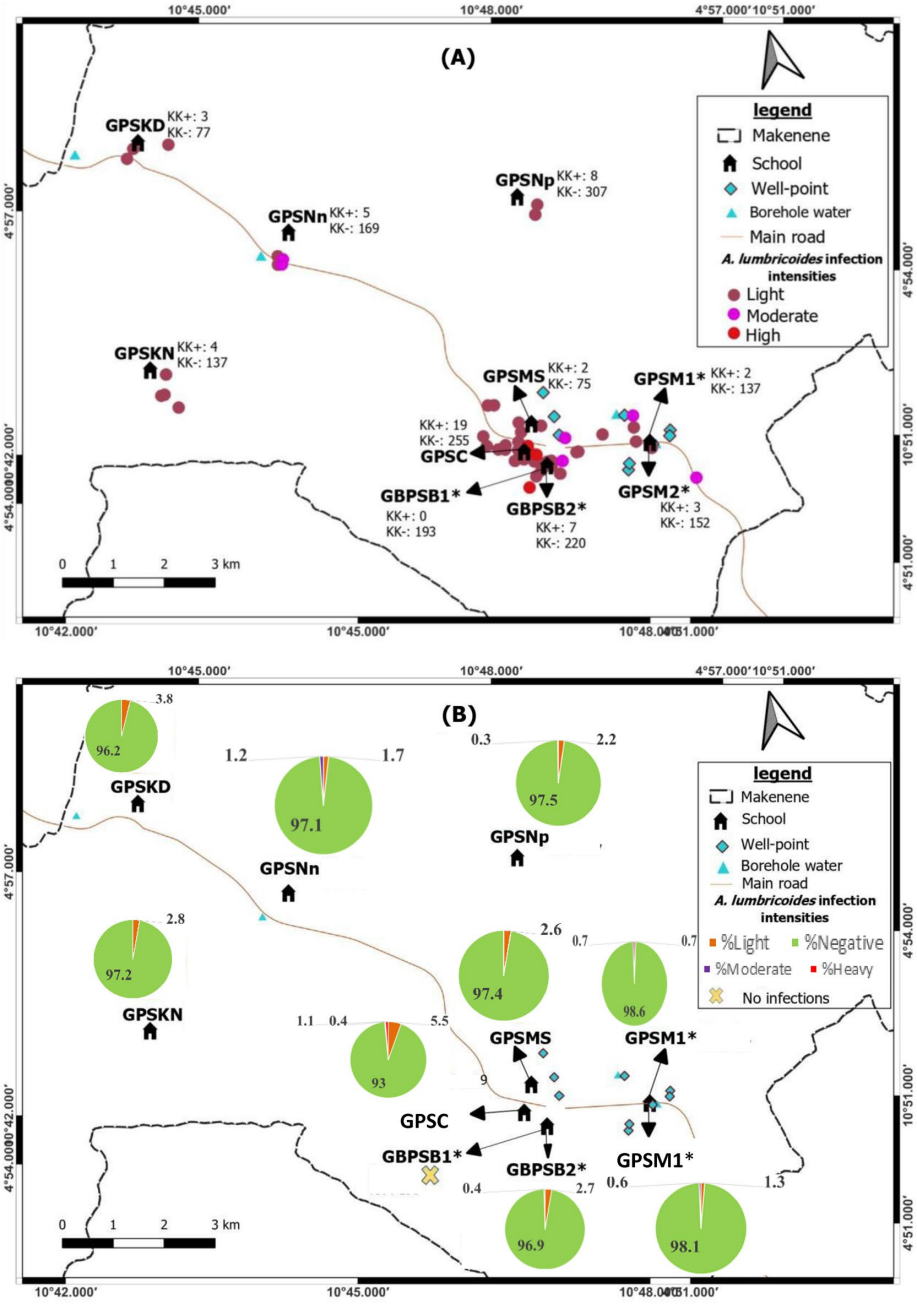
Map showing spatial distribution of *Ascaris lumbricoides* infections (**A**) and its infection intensities (**B**) according to schools (*both schools are hosted on the same building). GBPSB1: Government Bilingual Primary School of Baloua Group 1; GBPSB2: Government Bilingual Primary School of Baloua Group 2; GPSM1: Government Primary School of Makenene Group 1; GPSM2: Government Primary School of Makenene Group 2; GPSNn: Government Primary School of Nyokon; GPSKD: Government Primary School of Kinding Nde; GPSKN: Government Primary School of Kinding-Ndjabi; GPSMS: Government Primary School of Mock-Sud; GPSC: Government Primary School of Carrière; GPSNp: Government Primary School of Ngokop.

**Figure 4:**
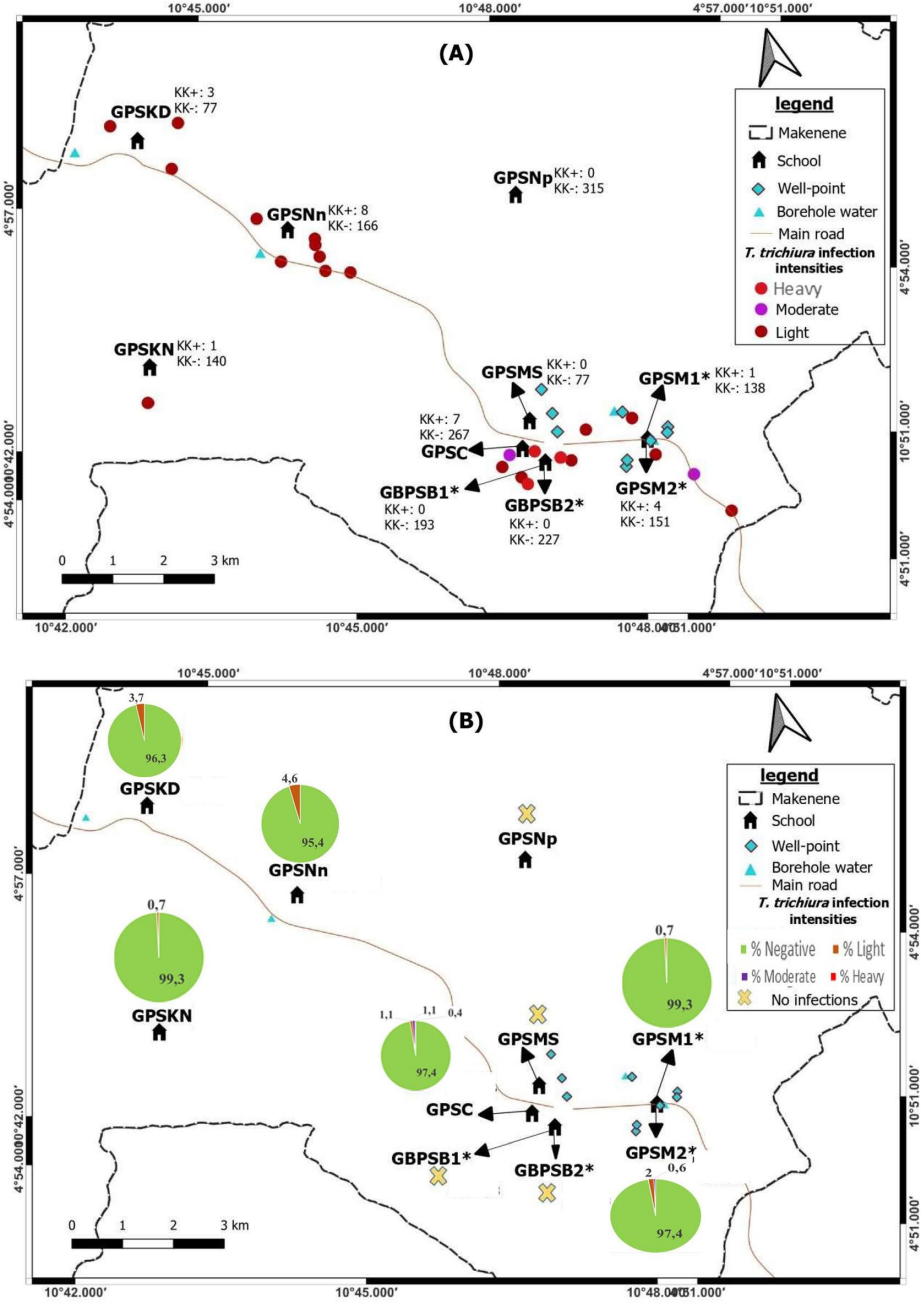
Map showing spatial distribution of *Trichuris trichiura* infections (**A**) and its infection intensities (**B**) according to schools (*both schools are hosted on the same building). GBPSB1: Government Bilingual Primary School of Baloua Group 1; GBPSB2: Government Bilingual Primary School of Baloua Group 2; GPSM1: Government Primary School of Makenene Group 1; GPSM2: Government Primary School of Makenene Group 2; GPSNn: Government Primary School of Nyokon; GPSKD: Government Primary School of Kinding Nde; GPSKN: Government Primary School of Kinding-Ndjabi; GPSMS: Government Primary School of Mock-Sud; GPSC: Government Primary School of Carrière; GPSNp: Government Primary School of Ngokop.

**Figure 5:**
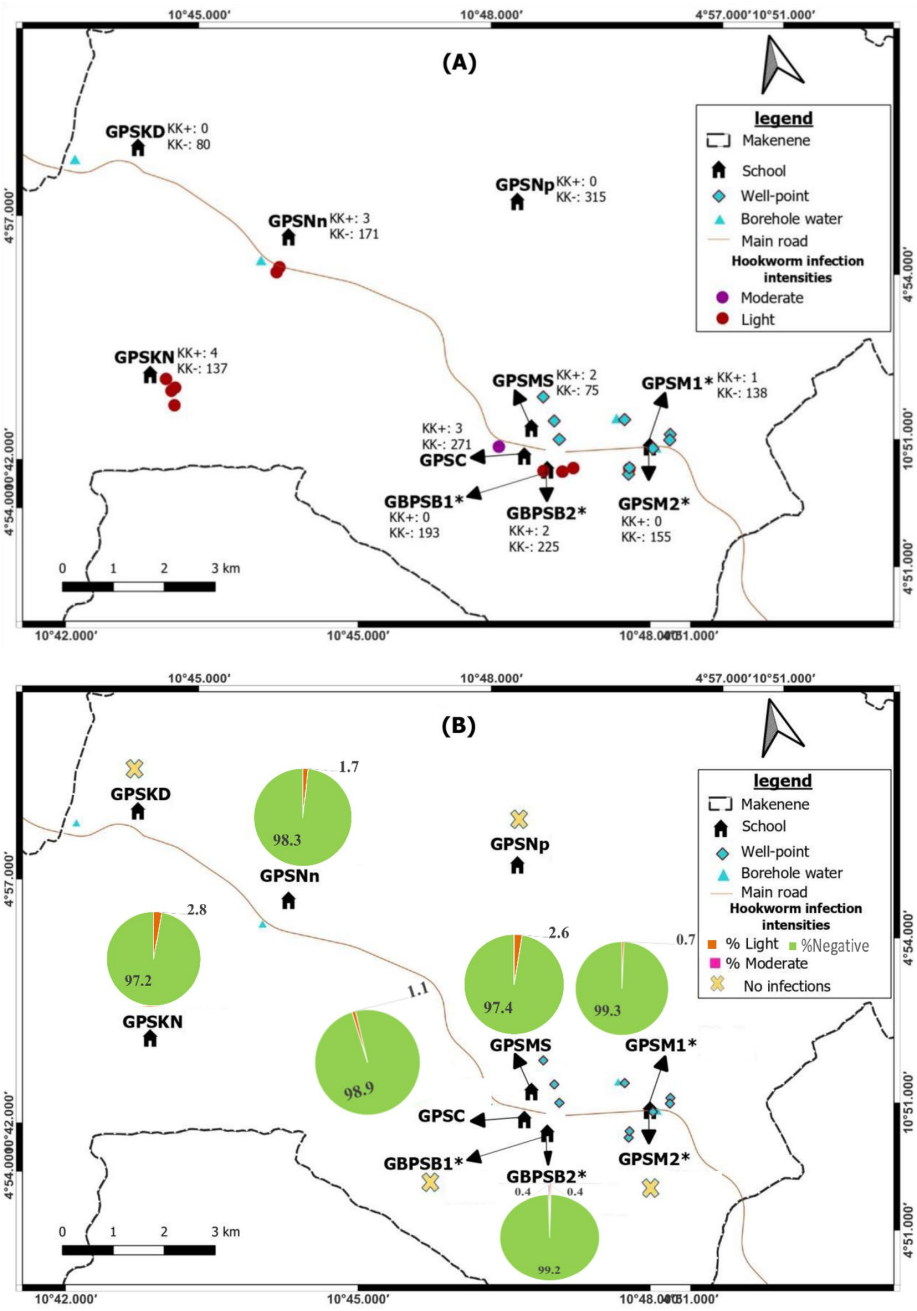
Map showing spatial distribution of hookworm infections (**A**) and its infection intensities (**B**) according to schools (*both schools are hosted on the same building). GBPSB1: Government Bilingual Primary School of Baloua Group 1; GBPSB2: Government Bilingual Primary School of Baloua Group 2; GPSM1: Government Primary School of Makenene Group 1; GPSM2: Government Primary School of Makenene Group 2; GPSNn: Government Primary School of Nyokon; GPSKD: Government Primary School of Kinding Nde; GPSKN: Government Primary School of Kinding-Ndjabi; GPSMS: Government Primary School of Mock-Sud; GPSC: Government Primary School of Carrière; GPSNp: Government Primary School of Ngokop.

The prevalence of soil-transmitted helminths vary according to species and sub-districts. In Ngokop sub-district, no child was found with egg of hookworm or *T. trichiura* while *A. lumbricoides* eggs were recorded in children from all sub-districts and all schools except the Government Bilingual Primary School of Baloua Group 1 (Figs. 2, 3, 4 and 5). Compared to *T. trichiura* and hookworm, *A. lumbricoides* infections seem to be widely distributed in schools and sub-districts (Fig. 3). Hookworm infections were recorded in children from all sub-districts except those from Kinding Nde and Ngokop (Fig. 5). The distribution of soil-transmitted helminth species in schools reflects the general situation recorded in sub-districts. Schools reporting various soil-transmitted helminth species were located in sub-districts recording identical profiles of parasite species.

Regarding other factors that were recorded in different sub-districts, the presence of wells and points for borehole water were positioned on different maps (Figs. 2, 3, 4 and 5). Although the geographical localization of toilet in poor hygienic conditions were not indicated in different figures, most of them were recorded in sub-districts like Mock-Sud, Baloua and Carrière.

### Spatial distribution of the infection intensities of each species of soil-transmitted helminths

Children carrying heavy infection intensities of soil-transmitted helminths were clustered in villages Baloua and Carriere (Fig. 2) while those harbouring light infection intensities were widely distributed in different sub-districts. Heavy infection of *A. lumbricoides* and *T. trichiura* were recorded in children from schools located at Carriere (Figs. 3 and 4). Moderate infection intensities of *A. lumbricoides, T. trichiura* and hookworms were recorded in children from schools of Carriere, Makenene and Baloua (Figs. 3 and 4) while light infection intensities were found in children from schools of all sub-districts (Figs. 3, 4 and 5).

## Discussion

Micro-mapping of soil-transmitted helminth infections and their intensities appears as key determinant for monitoring control measures that must lead to the WHO goal of eliminating soil-transmitted helminth infections as a public health problem by 2030. The presence of *A. lumbricoides, T. trichiura* and hookworms eggs confirms previous results and highlights the fact that soil-transmitted helminth infections still remain present although with low prevalence in sub-districts of Makenene after several years of PC^14,25–27^. The prevalence of 4.8% recorded in the present study is lower than 25.1% and 80% obtained eight (after the implementation of PC) and 19 (before the implementation of PC) years ago in the Centre Region of Cameroon^14,22^. This low prevalence can be considered as a great achievement for the National program for control of schistosomiasis and STH infections^14^. Our results are in agreement with those reporting the reduction of *A. lumbricoides* and *T. trichiura* prevalence in some endemic areas of Cameroon after the implementation of PC in 2007^14^. Although Mebendazole and Albendazole commonly used by other national control programs against other infectious agents such lymphatic filariasis can contribute to the reduction STH prevalence in some settings, this probability must be excluded because none of these anthelmintic drugs has been massively administered to inhabitants of Makenene, except by the national program for control of schistosomiasis and STH infections. The reduction of STH prevalence reported in the present study could be partially explained by the implementation of PC in which anthelmintic drugs have been annually and massively distributed to SAC^14^.

It could also be explained by the improvement of socioeconomic and environmental conditions in some ecological settings through land sanitation and construction of drinking water points. In some sub-districts of Makenene for instance, providing water of good quality through the construction of wells and water pumps by a non-governmental organization “Good-neighbor” has improved living conditions. This has probably impacted inhabitants’ behavior and consequently, the transmission and the epidemiology of soil-transmitted helminthiasis^14,28^. Results of the present study show that the locality of Makenene is moving toward the elimination STH infections.

The differences recorded in the prevalence and infection intensities of soil-transmitted helminth species could be related to variations of socioeconomic and ecological conditions in various sub-districts. The high prevalence of *A. lumbricoides* compared to other soil-transmitted helminth species does not agree with results of previous authors^22^ who reported, about 19 years ago, higher prevalence of *T. trichuria* in the same endemic area. This could be explained by the nature of eggs of different STH species. Compared to other STH species, eggs of *A. lumbricoides* can persist in the environment for several months by resisting to adverse bioecological conditions^29^. Although more sensitive to Mebendazole that have been commonly used in this locality^30,31^, the higher prevalence of *A. lumbricoides* could be explained by the fact that its adult worms produce more eggs compared to other STHs. Furthermore, we cannot rule out the probability of having eggs of *A. suum* because parasitological methods cannot differentiate them from those of *A. lumbricoides*. This hypothesis is strengthened by the fact that pigs raised by most inhabitants of different sub-districts move freely from one biotope to another. In such context, the possibility for *A.s suum* to be cross-transmitted to humans cannot be excluded. Although such transmission has been already demonstrated elsewhere^32^, no investigation has been undertaken in the locality of Makenene to detect *A. suum* and to further see if there is any cross-transmission between parasites from humans and pigs.

The low prevalence of hookworm infections obtained in this study can be explained by the transmission route, the sampled population and the short lifetime of infective larvae. As these larvae can stay in the soil for only 3–10 days, the probability of a treated child to be rapidly re-infected is low^33^. The low prevalence of hookworm could also be explained by the fact that very few inhabitants of different sub-districts walk barefoot (observation made during field surveys). Moreover, as hookworm prevalence and its infection intensities usually increases with age^34^, our data on hookworm were probably underestimated because only SAC were examined in this study. Results of the present study highlight the low transmission and the historical low endemicity or historical low prevalence of hookworm in this area; confirming results obtained 26 and 8 years ago in the same locality^14,22^.

The high prevalence of *A. lumbricoides* compared to *T. trichiura* contradicts previous results that report more *T. trichiura* (54.4% and 18.6%) than *A. lumbricoides* (37.6% and 10.5%)^14,22^. The discrepancies between these results could be explained by climate changes that have probably induced new environmental conditions that were less favourable for the development of eggs of some STH species. In fact, green vegetation provides shade which protects eggs from ultraviolet radiation and desiccation and also serves as a proxy for soil moisture which is needed for the development of *T. trichiura* eggs^35^. In addition, helminths’ ova can stop its development in the environment if they do not find favorable temperature and moisture conditions^36^. Taking into consideration the current bioclimatic conditions and those before the implementation of PC, it is obvious that the vegetation as well as the temperature have considerably changed. In such adverse bioclimatic conditions, eggs of *T. trichiura* are less resistant than those of *A. lumbricoides*^33,37^*;* justifying thus the current higher prevalence of *A. lumbricoides.*

Our results reporting no significant difference in the prevalence of soil-transmitted helminth infections according to sex or age corroborate those of previous studies^25,38,39^. These results could be explained by the fact that, independently of age and sex, SAC are probably exposed to the same contaminated environment. Sharing the same poor socioeconomic and environment conditions, boys and girls of all ages have the same probability of acquiring soil-transmitted helminth infections since these infections result from non-compliance of basic hygiene rules^26^.

Our results reporting co-infections of different STH species are in agreement with results of previous studies^26,40–44^. These co-infections may have some implications for the health of affected children. In fact, the synergistic effect of co-infecting parasites can affect the nutritional status of infected children by depriving them from important nutrients. As children harbouring single infections performed better in school than those with multiple infections^45^, control measures could be adjusted in sub-districts reporting children with co-infections and heavy infections. In addition to anthelmintic drugs, some nutrients could be provided as supplements to infected children of these sub-districts.

Although information regarding the number of months that passed after the last mass administration of anthelminthic drug has not been recorded, it is likely that most infected children and especially those carrying light infection intensities were recently re-infected, probably few months after taking anthelmintic drugs. This observation is strengthened by the low number of children harbouring heavy and moderate infection intensities. These results confirm the usefulness of PC in the reduction of STH prevalence. In addition to that, it is important to point out that improving socioeconomic, hygienic and environmental conditions could affect the transmission, the prevalence and the infection intensities of soil-transmitted helminths.

Compared to previous studies, the arithmetic means of the number of eggs per gram of stool for each soil-transmitted helminth species (7255 EPG for *A. lumbricoides*, 2900 EPG for *T. trichiura* and 298 EPG hookworms) were higher than those (1086.02 EPG, 113.04 EPG and 8.55 EPG respectively for *A. lumbricoides*, *T. trichiura* and hookworms) of previous studies^14^. Our results are difficult to explain if we take into consideration the low STH prevalence, the acceptability of anthelmintic drugs by people of this endemic locality, the easy accessibility to most sub-districts and the regular mass administration of anthelmintics in this locality. Nevertheless, we can speculate about the study design and the clustering of infected children. In the present study, all children of ten schools of eight sub-districts were examined while in previous ones, only four schools of each health district of the Region were involved. In addition, only 50 children were examined per school in previous studies. Mixing up the infection intensities of sub-districts having different levels of infections has generated the mean infection intensities for the entire region. This has probably led to an underestimation of the infection intensities in settings of high endemicity like some sub-districts of Makenene. This hypothesis is strengthened by the heterogeneous distribution of soil-transmitted helminth infections as well as their infection intensities in different sub-districts. This heterogeneous distribution was highlighted by the clustering of infected children and especially those carrying heavy infection intensities in some sub-districts. This clustering indicates not only the presence of high endemicity sub-districts where STH transmission is more important, but also the need of performing fine mapping of soil-transmitted helminth species. The high intensity of *T. trichiura* infection recorded in boys compared to girls is in agreement with results obtained by Bopda et al.^46^ in the Central Region of Cameroon. These results could be explained by the fact boys of different sub-districts of Makenene walk and play barefoot (field observations) and hence, are more exposed to *T. trichiura* eggs than girls. The clustering of children carrying heavy infection intensities could be explained by the lack of clean toilet and potable water points (wells and borehole water) around their houses or around environment where they live. In such environment, SAC are regularly in contact with contaminated environments. Being frequently in risky biotopes for playing, searching fruits and harvesting vegetables, SAC are more exposed and consequently, have a higher risk of acquiring soil-transmitted helminths. Moreover, the maps showing spatial distribution of soil-transmitted helminth infections reveal that residences of infected children were clustering around the Government Primary Schools of Carriere, the Government Bilingual Primary Schools of Baloua and the Government Primary school of Mock Sud. Located in sub-districts Carriere, Baloua and Mock-Sud, these sub-districts can be considered as the most risky ones. This hypothesis is strengthened by the identification of heavy infections of soil-transmitted helminth species in children of these sub-districts. Furthermore, the fact that water points with poor hygienic conditions were recorded in these sub-districts while potable water points (borehole water and wells) were scarce are additional arguments supporting high transmission of soil-transmitted helminths at Carriere, Baloua and Mock-Sud. These observations provide evidence for the presence of high endemicity sub-districts having transmission hot-spots for soil-transmitted helminths. The wide spatial distribution of children carrying low infection intensities could be explained by the scarcity of risky biotopes in sub-districts of low endemicity.

Results of fine mapping have shown that, within the same endemic area, the prevalence of each soil-transmitted helminth species as well as its infection intensities vary significantly according to sub-districts. These variations have some implications in the redesigning of control measures that must be implemented to achieve the elimination soil-transmitted helminth infections. As already reported by previous authors^47–49^, the variations observed in the STH prevalence suggest adaptions of control measures for each epidemiological setting. In sub-districts where the transmission and the prevalence of STH infections was high or in sub-districts escaping mass deworming program, control operations must be boosted by treating not only SAC, but also adults. In addition to that, two rounds of treatment could be implemented by alternating use of Mebendazole and Albendazole from one deworming round to another because some STH species could be escaping mass deworming program when only one anthelmintic drug is used^50^. Adapting and focusing control measures will reduce the efforts and the costs of control operations. Performing such activities will obviously reduce the prevalence and infection intensities of soil-transmitted helminths and subsequently, the morbidity associated to these parasitic diseases.

The present study has some limitations because any information regarding socioeconomic data, access to safe water, availability and characteristics of sanitation facilities at the local schools and in sub-districts have not been collected. Moreover, hygiene has not been considered in the analyses because data on hygiene practices have not been collected during field surveys. The relationship between these data and the prevalence and/or the infection intensities of different STH species cannot therefore be assessed although all these factors are able to influence the transmission of different STH species and subsequently, the epidemiology and the control of STH infections.

## Conclusion

This study highlighted variations in the prevalence and infection intensities of soil-transmitted helminth species according to sub-districts. It also highlighted the need to perform micro scale mapping for the identification and localization of sub-districts of high endemicity and hot-spot transmission sites for targeted control operations against soil-transmitted helminthiasis. Achieving the elimination STH infections requires adjusting control measures according to the epidemiological situation in each sub-district. In high endemicity sub-districts, applying targeted control measures could lead to soil-transmitted helminthiasis elimination.

## Data Availability

All data generated and/or analyzed during this study are included in the article.
